# Supplementary material for: scTimeBench: a streamlined benchmarking platform for single-cell time-series analysis
Source: Bioinformatics. 2026 Aug 21;42(Suppl 2):btag414. doi: 10.1093/bioinformatics/btag414 (PMC13501279; doi:10.1093/bioinformatics/btag414)
Supplement: Supplementary file 1 [file btag414_supplementary_data.pdf]

Supplementary Materials

S1 Pseudotime inference methodology

To enable pseudotime-based analyses, we employed *Diffusion Pseudotime* as implemented in the scanpy framework (Wolf et al., 2018; Haghverdi et al., 2016). This method infers pseudotime as a continuous metric of cellular progression by calculating the relative distances of cell embeddings across a low-dimensional space of a given scRNA-seq dataset. Diffusion pseudotime requires a designated root to anchor the trajectory; we consistently defined this starting point as the first cell captured of the earliest time point. The resulting pseudotime of any given cell represents its distance from this root within the inferred low-dimensional space. We then discretize the data into n pseudotime bins ($n = 15$ for the Garcia-Alonso and Suo datasets, and $n = 10$ for the Ma dataset). Cells were assigned to the nearest bin while ensuring an approximately uniform distribution of cells across each interval. The resulting labels provide an approximation of cellular progression relative to the progenitor cell.

S2 Supplementary Tables

Table S1 Summary of benchmark datasets including organism, tissue, and feature counts.

Dataset	Organism	Tissue	Cells	#tps	Genes	Source
ZB	Zebrafish	Embryo	38,731	12	2,000	Farrell et al. (2018)
DR	Drosophila	Embryo	27,386	11	2,000	Calderon et al. (2022)
MEF	Mouse	Embryo	236,285	19	2,000	Schiebinger et al. (2019)
Ma (Forecast)	Human	Pancreas	76,806	8	29,534	Ma et al. (2023)
Olaniru	Human	Pancreas	26,220	7	29,534	Olaniru et al. (2023)
Ma+Olaniru	Human	Pancreas	103,026	15	29,534	Ma et al. (2023); Olaniru et al. (2023)
Garcia-Al.	Human	Gonad	10,993[1]	15	27,950	Garcia-Alonso et al. (2022)
Ma (Lineage)	Human	Pancreas	6,312[1]	8	29,534	Ma et al. (2023)
Suo	Human	B cells	65,413[1]	11	33,538	Suo et al. (2022)

[1]Cell counts after filtering for cell types which exist in the reference lineage.

Table S2 Supplemental csv file for metric-specific results for forecast accuracy tasks.

Table S3 Supplemental csv file for ARI-specific embedding coherence results.

Table S4 Supplemental csv file for Entropy-specific embedding coherence results.

Table S5 Cell Typist average 3-fold cross-validation scores. We used CellTypist to classify the projected gene expression for generative models.

Dataset	Accuracy	Weighted F1-Score
Garcia-Alonso	0.907	0.905
Ma	0.902	0.899
Suo	0.899	0.897

Table S6 Weighted average entropy of cell types per timepoint

Dataset	Observed Time Entropy	Pseudotime Entropy
Garcia-Alonso	1.203	0.760
Ma	0.651	0.702
Suo	1.967	1.646

Table S7 Lineage fidelity metrics in the single-step setting across methods and datasets.

Time Type	Method	Garcia-Alonso			Ma			Suo		
		Jaccard	AUPRC	AUROC	Jaccard	AUPRC	AUROC	Jaccard	AUPRC	AUROC
Observed Time	CellMNN	0.167	0.227	0.632	0.500	0.383	0.769	**0.091**	0.156	0.670
	Correlation	**0.238**	0.262	0.710	0.250	0.529	0.692	**0.091**	0.270	0.679
	MIOFlow	0.179	0.159	0.510	0.273	0.313	0.641	**0.091**	0.213	0.599
	Moscot	0.208	0.367	0.645	0.333	0.778	0.846	**0.091**	**0.271**	0.677
	PISDE	0.167	0.151	0.535	0.250	0.236	0.590	**0.091**	0.083	0.455
	PRESCIENT	0.167	0.190	0.535	0.375	0.681	0.846	**0.091**	0.111	0.566
	WOT	0.227	**0.370**	0.665	0.500	0.833	0.923	–	–	–
	OT-CFM	0.208	0.367	**0.748**	**0.750**	**0.917**	**0.974**	**0.091**	0.251	**0.696**
	scIMF	0.179	0.197	0.535	0.300	0.239	0.538	**0.091**	0.122	0.608
	scNODE	0.167	0.146	0.471	0.250	0.340	0.641	**0.091**	0.149	0.613
Pseudotime	CellMNN	0.167	0.216	0.619	0.500	**0.667**	0.872	0.091	0.143	0.666
	Correlation	0.294	0.294	0.768	0.429	0.443	0.795	0.091	0.223	0.696
	MIOFlow	0.250	0.287	0.735	–	–	–	–	–	–
	Moscot	0.417	0.536	0.871	**0.750**	0.639	**0.923**	**0.105**	0.223	0.683
	PISDE	0.167	0.151	0.523	0.429	0.421	0.782	0.091	0.104	0.537
	PRESCIENT	0.312	0.424	0.774	0.375	0.458	0.795	0.091	0.169	0.616
	WOT	0.385	0.576	0.877	**0.750**	0.639	**0.923**	–	–	–
	OT-CFM	**0.714**	**0.826**	**0.968**	**0.750**	0.639	**0.923**	0.091	**0.227**	**0.718**
	scIMF	0.312	0.274	0.761	0.500	0.411	0.795	0.091	0.150	0.667
	scNODE	0.167	0.185	0.581	0.250	0.257	0.577	0.100	0.190	0.561

Table S8 Lineage fidelity metrics in the multi-step setting across methods and datasets.

Time Type	Method	Garcia-Alonso			Ma			Suo		
		Jaccard	AUPRC	AUROC	Jaccard	AUPRC	AUROC	Jaccard	AUPRC	AUROC
Observed Time	CellMNN	**0.500**	0.436	0.281	0.692	0.511	0.444	0.512	0.551	0.512
	Correlation	**0.500**	0.401	0.173	0.562	0.627	0.429	0.512	0.576	0.517
	MIOFlow	**0.500**	0.375	0.198	0.562	0.456	0.206	0.512	0.584	0.506
	Moscot	**0.500**	0.465	0.299	0.778	0.644	0.429	0.625	0.585	0.554
	PISDE	**0.500**	**0.483**	**0.417**	0.562	0.586	0.540	0.512	0.512	0.415
	PRESCIENT	**0.500**	0.426	0.338	0.692	0.651	0.460	0.512	0.539	0.459
	WOT	**0.500**	0.440	0.241	0.778	0.727	0.603	–	–	–
	OT-CFM	**0.500**	0.441	0.255	**1.000**	**0.806**	**0.651**	**0.667**	**0.614**	**0.606**
	scIMF	**0.500**	0.389	0.182	0.562	0.428	0.127	0.512	0.509	0.412
	scNODE	**0.500**	0.448	0.228	0.562	0.531	0.365	0.512	0.540	0.517
Pseudotime	CellMNN	0.500	0.419	0.290	0.692	0.605	0.476	0.512	0.557	0.531
	Correlation	0.500	0.408	0.255	0.692	0.538	0.476	0.512	0.593	0.545
	MIOFlow	0.500	0.432	0.321	–	–	–	–	–	–
	Moscot	0.720	0.522	0.444	**0.900**	0.599	0.508	0.598	0.589	0.568
	PISDE	0.500	0.483	0.410	0.818	0.607	**0.587**	0.512	0.511	0.423
	PRESCIENT	0.720	0.649	**0.546**	0.692	**0.621**	0.540	0.512	0.590	0.544
	WOT	0.720	0.549	0.465	**0.900**	0.599	0.508	–	–	–
	OT-CFM	**0.947**	**0.661**	0.509	**0.900**	0.616	0.540	**0.636**	**0.627**	**0.623**
	scIMF	0.500	0.400	0.272	0.692	0.504	0.397	0.512	0.553	0.463
	scNODE	0.500	0.436	0.349	0.562	0.480	0.325	0.512	0.598	0.598

S3 Supplementary Figures

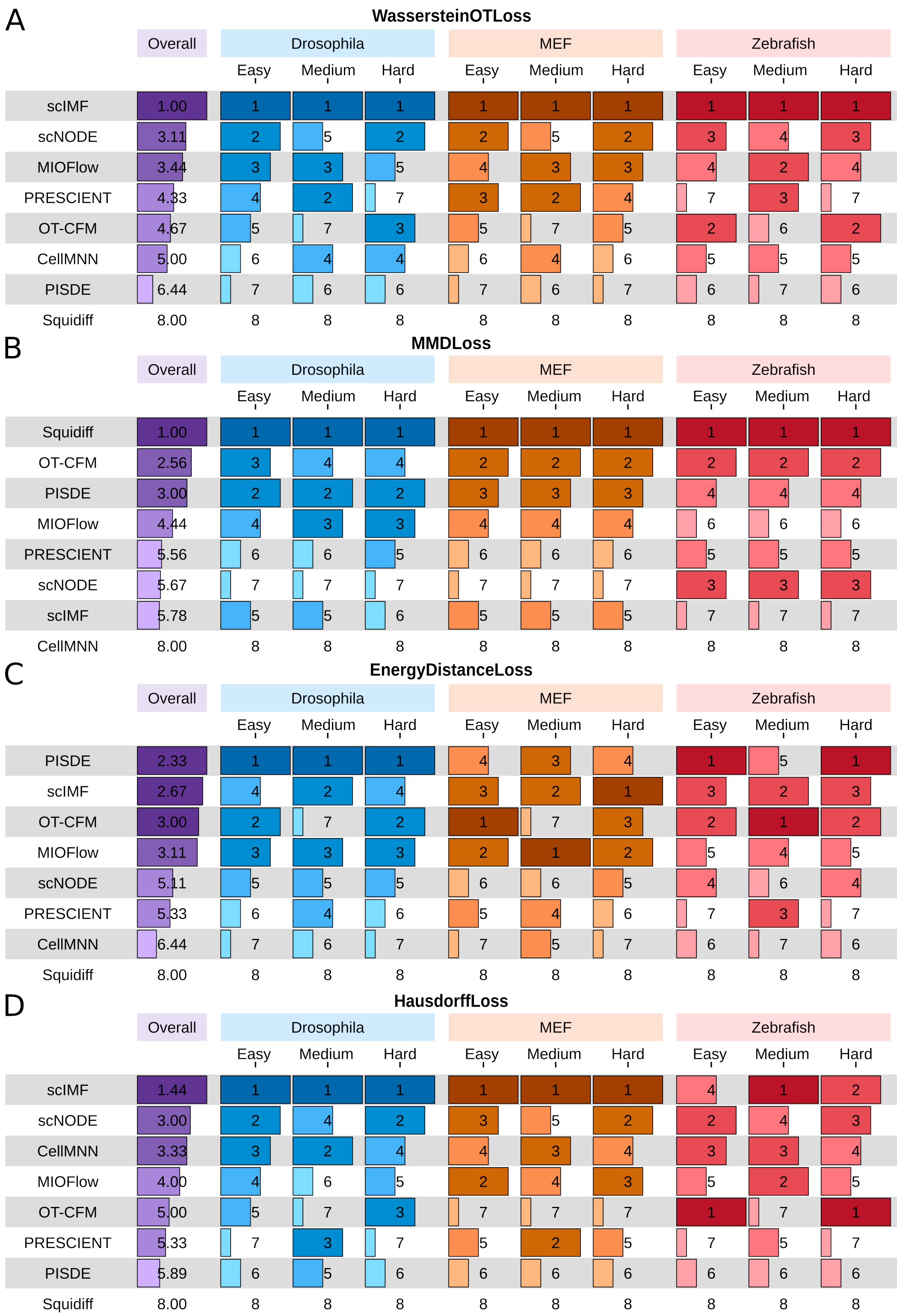

Figure S1 Average rank metric for HVG datasets in terms of Wasserstein distance (A), Gaussian MMD (B), Energy Distance MMD (C) and Hausdorff Loss (D).

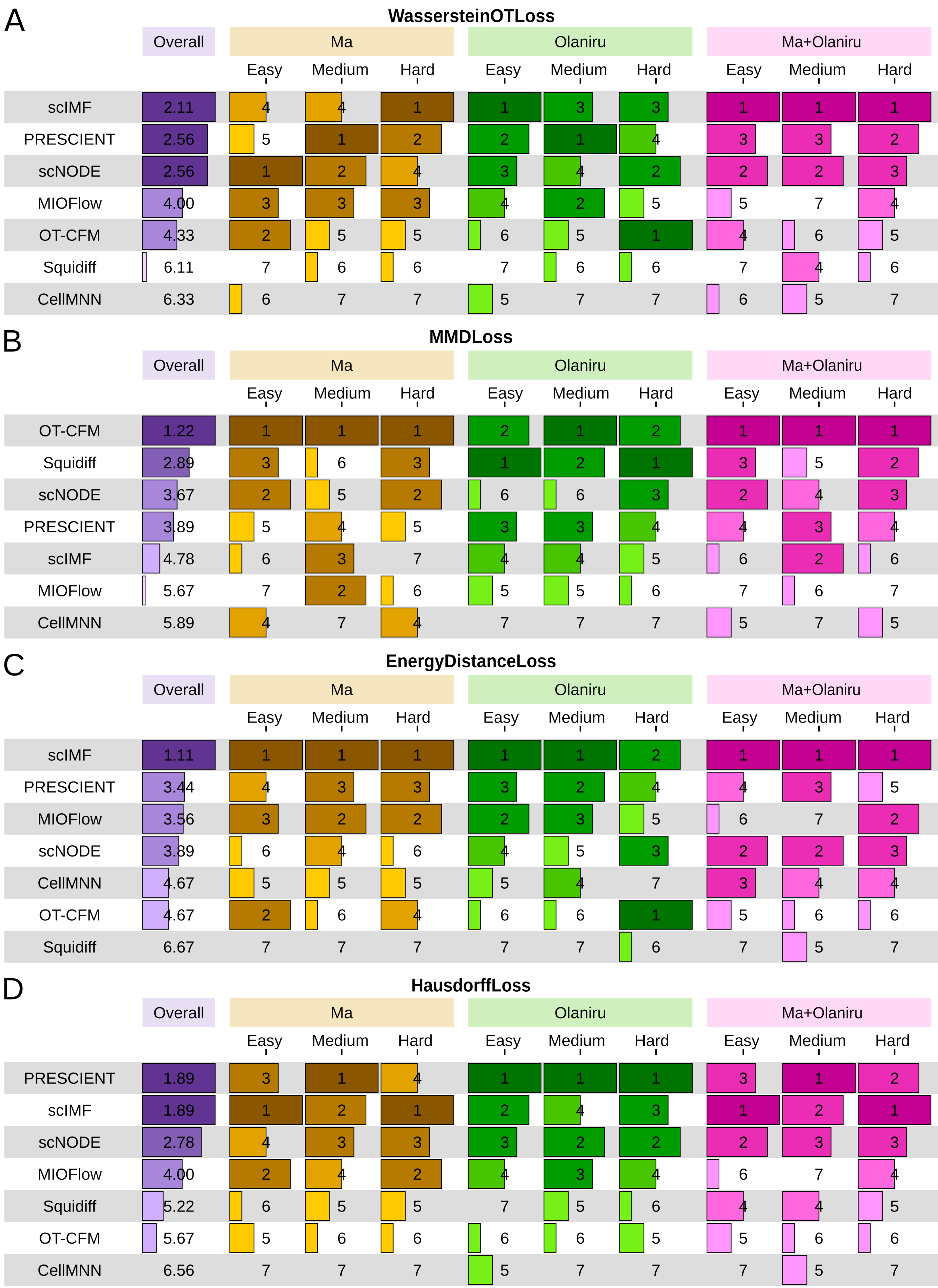

Figure S2 Average rank metric for pancreas development datasets in terms of Wasserstein distance (A), Gaussian MMD (B), Energy Distance MMD (C) and Hausdorff Loss (D).

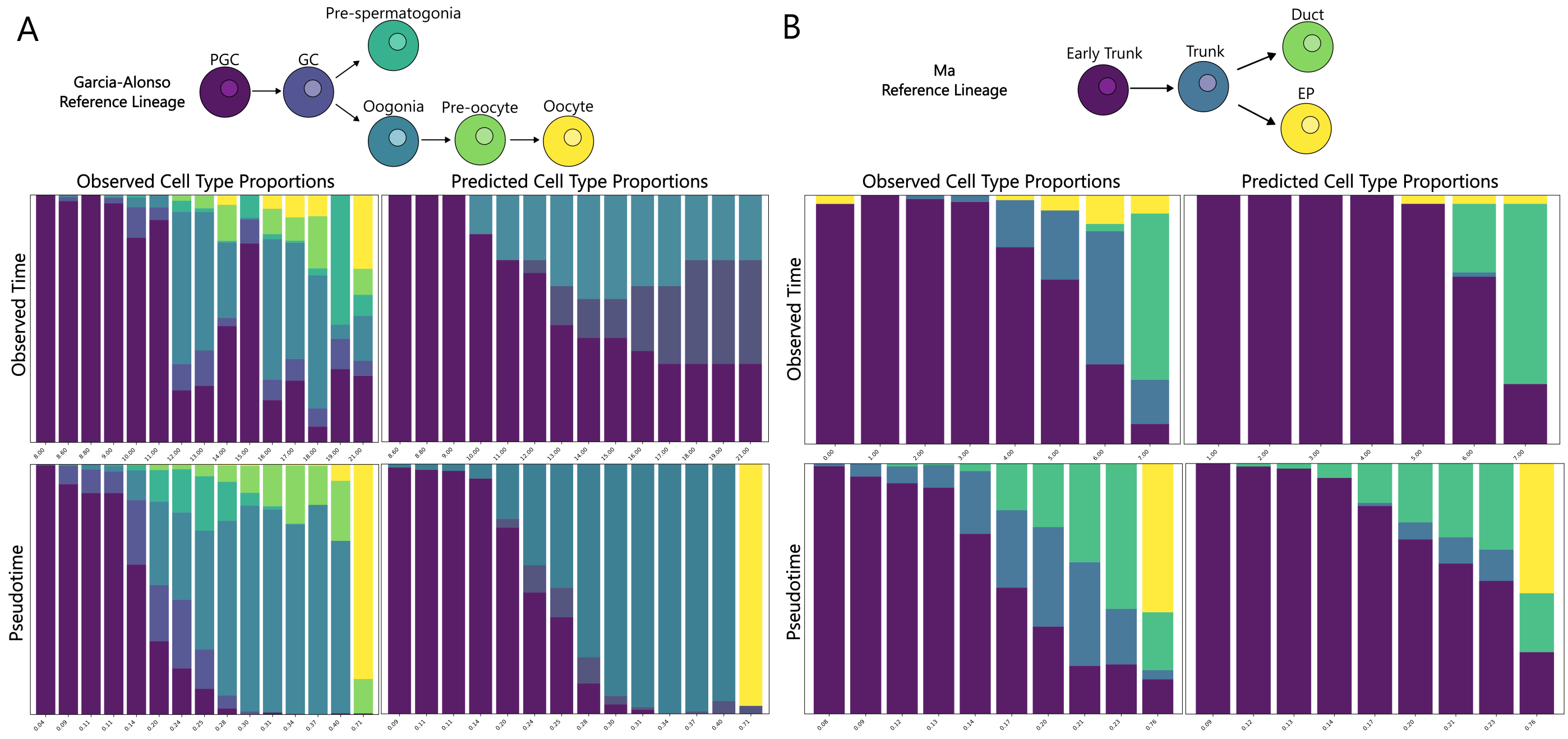

Figure S3 Pseudotime vs. Observed time cell-type proportions for observed and predicted Garcia-Alonso (A) and Ma (B) data. scNODE cell-type proportions were generated by projecting cells from the initial observed timepoint forward to all subsequent timepoints.

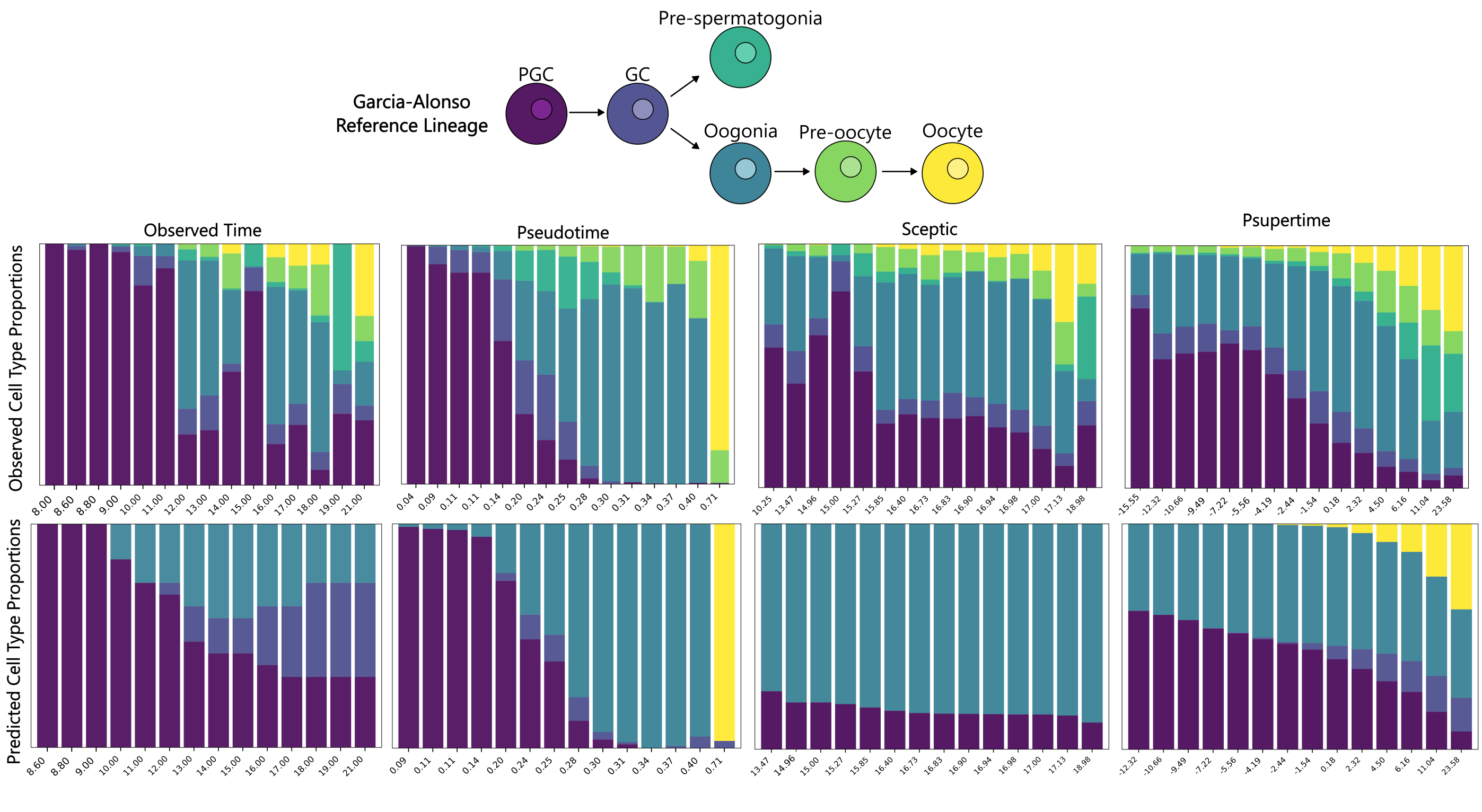

Figure S4 Predicted and observed cell-type proportions for various temporal labels (observed time, pseudotime, Sceptic, and Psupertime) of Garcia-Alonso data (Li et al., 2025; Macnair et al., 2022). scNODE cell-type proportions were generated by projecting cells from the initial observed timepoint forward to all subsequent timepoints.

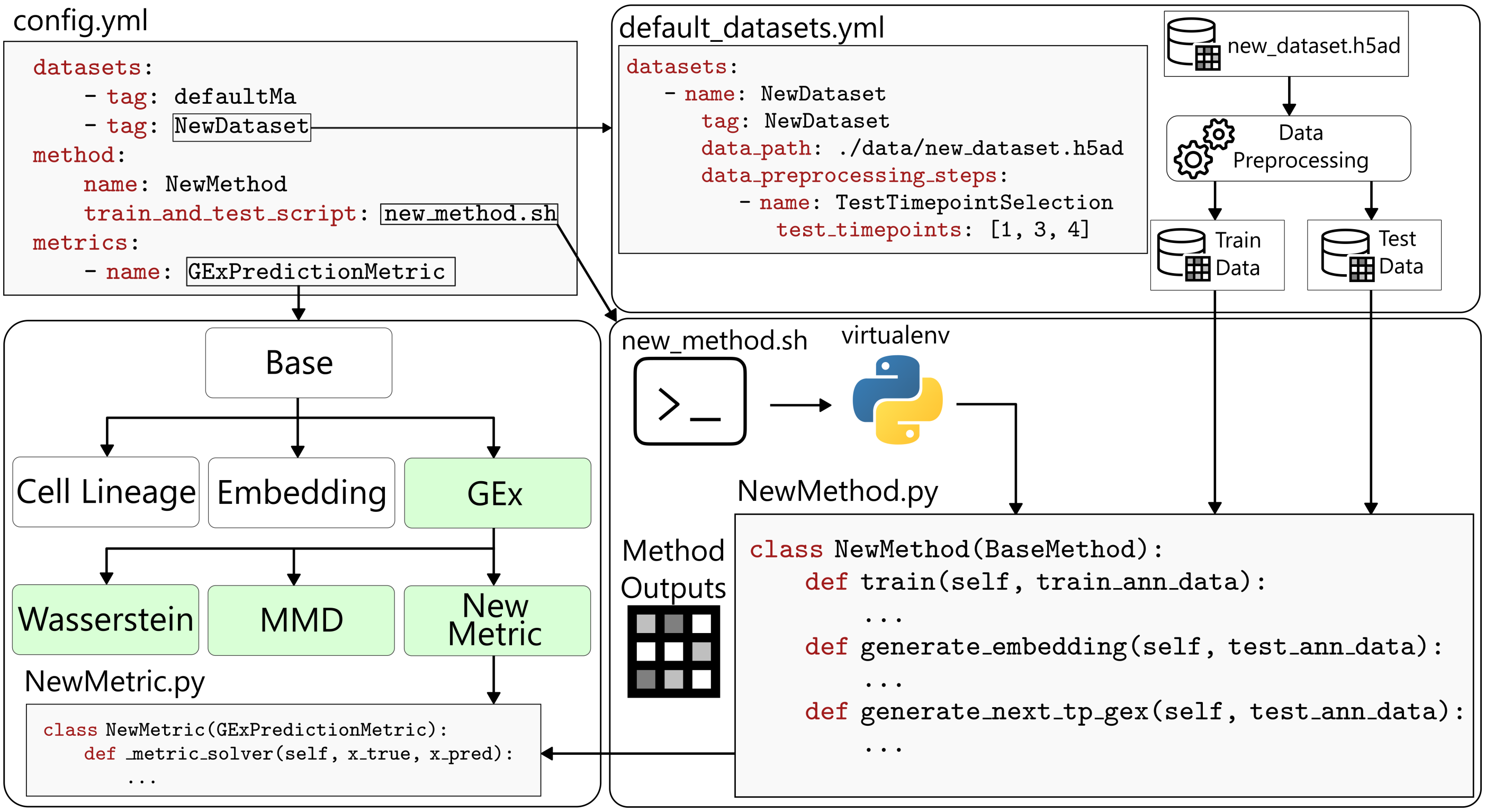

Figure S5 `scTimeBench` **pipeline.** The framework utilizes a centralized configuration file to orchestrate the execution of datasets, models, and metrics. The user defines an experiment's datasets through a list of tags, each of which is associated with a particular dataset configuration. The user also defines the method used by providing a training and evaluation script alongside any required training hyperparameters. This method is trained and evaluated on the datasets to produce the specified metrics. More information can be found in the online documentation: https://li-lab-mcgill.github.io/scTimeBench/. **Dataset Integration:** New datasets require a standard Anndata object (.h5ad) file and a corresponding configuration entry which specify preprocessing routines for consistent train-test splits. **Model Integration:** The platform supports diverse model architectures, requiring users to provide (1) a shell script that specifies a virtual environment and (2) a Python implementation of the model training and output generation. **Metric Integration:** Metrics are organized into a class-based hierarchy to streamline comparative analysis. For example, all Gene Expression (GEx)/forecast accuracy metrics inherit a unified interface, requiring only the definition of an evaluation function that accepts observed GEx X^t and predicted GEx $\hat{X}^{t+1}$. Specifying a parent class (e.g. GExPredictionMetric) in the configuration will run all existing GEx metrics, including any user-defined custom metrics.

Supplementary Table S2: Forecast Accuracy results.

Method	Dataset	Metric	Result	Task
CellMNN	Drosophila	EnergyDistanceLoss	0.003889957	Easy
CellMNN	MEF	EnergyDistanceLoss	0.003251428	Easy
CellMNN	Ma	EnergyDistanceLoss	0.001441732	Easy
CellMNN	MaOlaniru	EnergyDistanceLoss	0.002723711	Easy
CellMNN	Olaniru	EnergyDistanceLoss	0.003598932	Easy
CellMNN	Zebrafish	EnergyDistanceLoss	0.00439347	Easy
CellMNN	Drosophila	EnergyDistanceLoss	0.003315826	Hard
CellMNN	MEF	EnergyDistanceLoss	0.002005826	Hard
CellMNN	Ma	EnergyDistanceLoss	0.002335427	Hard
CellMNN	MaOlaniru	EnergyDistanceLoss	0.004559044	Hard
CellMNN	Olaniru	EnergyDistanceLoss	1.361836591	Hard
CellMNN	Zebrafish	EnergyDistanceLoss	0.004298922	Hard
CellMNN	Drosophila	EnergyDistanceLoss	0.004522222	Medium
CellMNN	MEF	EnergyDistanceLoss	0.001964147	Medium
CellMNN	Ma	EnergyDistanceLoss	0.002867059	Medium
CellMNN	MaOlaniru	EnergyDistanceLoss	0.012666717	Medium
CellMNN	Olaniru	EnergyDistanceLoss	0.00523501	Medium
CellMNN	Zebrafish	EnergyDistanceLoss	0.008838527	Medium
CellMNN	Drosophila	HausdorffLoss	44.49737723	Easy
CellMNN	MEF	HausdorffLoss	24.89288194	Easy
CellMNN	Ma	HausdorffLoss	513042.5099	Easy
CellMNN	MaOlaniru	HausdorffLoss	1183677.117	Easy
CellMNN	Olaniru	HausdorffLoss	6759.325181	Easy
CellMNN	Zebrafish	HausdorffLoss	47.49694971	Easy
CellMNN	Drosophila	HausdorffLoss	44.60980908	Hard
CellMNN	MEF	HausdorffLoss	24.67573664	Hard
CellMNN	Ma	HausdorffLoss	2456754.058	Hard
CellMNN	MaOlaniru	HausdorffLoss	4920423.99	Hard
CellMNN	Olaniru	HausdorffLoss	9284681934	Hard
CellMNN	Zebrafish	HausdorffLoss	47.65203046	Hard
CellMNN	Drosophila	HausdorffLoss	46.66725973	Medium
CellMNN	MEF	HausdorffLoss	28.35944798	Medium
CellMNN	Ma	HausdorffLoss	985071074	Medium
CellMNN	MaOlaniru	HausdorffLoss	5021672.188	Medium
CellMNN	Olaniru	HausdorffLoss	4449426.083	Medium
CellMNN	Zebrafish	HausdorffLoss	51.19047672	Medium
CellMNN	Drosophila	MMDLoss	1.51E-06	Easy
CellMNN	MEF	MMDLoss	6.64E-06	Easy
CellMNN	Ma	MMDLoss	3.09E-09	Easy
CellMNN	MaOlaniru	MMDLoss	6.16E-09	Easy
CellMNN	Olaniru	MMDLoss	1.10E-08	Easy
CellMNN	Zebrafish	MMDLoss	1.63E-06	Easy

CellMNN	Drosophila	MMDLoss	8.36E-07	Hard
CellMNN	MEF	MMDLoss	7.66E-06	Hard
CellMNN	Ma	MMDLoss	2.84E-09	Hard
CellMNN	MaOlaniru	MMDLoss	7.63E-09	Hard
CellMNN	Olaniru	MMDLoss	Inf	Hard
CellMNN	Zebrafish	MMDLoss	1.35E-06	Hard
CellMNN	Drosophila	MMDLoss	1.16E-06	Medium
CellMNN	MEF	MMDLoss	7.44E-06	Medium
CellMNN	Ma	MMDLoss	5.29E-09	Medium
CellMNN	MaOlaniru	MMDLoss	1.06E-08	Medium
CellMNN	Olaniru	MMDLoss	1.16E-08	Medium
CellMNN	Zebrafish	MMDLoss	2.17E-06	Medium
CellMNN	Drosophila	WassersteinOTLoss	0.293576251	Easy
CellMNN	MEF	WassersteinOTLoss	0.09250493	Easy
CellMNN	Ma	WassersteinOTLoss	843.3566633	Easy
CellMNN	MaOlaniru	WassersteinOTLoss	4409.140013	Easy
CellMNN	Olaniru	WassersteinOTLoss	34.5267946	Easy
CellMNN	Zebrafish	WassersteinOTLoss	0.306024004	Easy
CellMNN	Drosophila	WassersteinOTLoss	0.289626292	Hard
CellMNN	MEF	WassersteinOTLoss	0.068620769	Hard
CellMNN	Ma	WassersteinOTLoss	40078.65863	Hard
CellMNN	MaOlaniru	WassersteinOTLoss	184208.0645	Hard
CellMNN	Olaniru	WassersteinOTLoss	5.38527E+11	Hard
CellMNN	Zebrafish	WassersteinOTLoss	0.327781129	Hard
CellMNN	Drosophila	WassersteinOTLoss	0.372880197	Medium
CellMNN	MEF	WassersteinOTLoss	0.077438581	Medium
CellMNN	Ma	WassersteinOTLoss	3781357641	Medium
CellMNN	MaOlaniru	WassersteinOTLoss	444724.9588	Medium
CellMNN	Olaniru	WassersteinOTLoss	297441.5774	Medium
CellMNN	Zebrafish	WassersteinOTLoss	0.584703153	Medium
MIOFlow	Drosophila	EnergyDistanceLoss	0.001779724	Easy
MIOFlow	MEF	EnergyDistanceLoss	0.000666953	Easy
MIOFlow	Ma	EnergyDistanceLoss	0.000850983	Easy
MIOFlow	MaOlaniru	EnergyDistanceLoss	0.013937141	Easy
MIOFlow	Olaniru	EnergyDistanceLoss	0.002005053	Easy
MIOFlow	Zebrafish	EnergyDistanceLoss	0.00278828	Easy
MIOFlow	Drosophila	EnergyDistanceLoss	0.001566001	Hard
MIOFlow	MEF	EnergyDistanceLoss	0.000572216	Hard
MIOFlow	Ma	EnergyDistanceLoss	0.000972177	Hard
MIOFlow	MaOlaniru	EnergyDistanceLoss	0.003041507	Hard
MIOFlow	Olaniru	EnergyDistanceLoss	0.004831413	Hard
MIOFlow	Zebrafish	EnergyDistanceLoss	0.00300341	Hard
MIOFlow	Drosophila	EnergyDistanceLoss	0.002294845	Medium
MIOFlow	MEF	EnergyDistanceLoss	0.000496421	Medium

MIOFlow	Ma	EnergyDistanceLoss	0.001531397	Medium
MIOFlow	MaOlaniru	EnergyDistanceLoss	6.896090525	Medium
MIOFlow	Olaniru	EnergyDistanceLoss	0.004033048	Medium
MIOFlow	Zebrafish	EnergyDistanceLoss	0.003075708	Medium
MIOFlow	Drosophila	HausdorffLoss	44.83819455	Easy
MIOFlow	MEF	HausdorffLoss	23.44934562	Easy
MIOFlow	Ma	HausdorffLoss	9357.092141	Easy
MIOFlow	MaOlaniru	HausdorffLoss	46147.68784	Easy
MIOFlow	Olaniru	HausdorffLoss	6655.676882	Easy
MIOFlow	Zebrafish	HausdorffLoss	49.60393774	Easy
MIOFlow	Drosophila	HausdorffLoss	48.90101251	Hard
MIOFlow	MEF	HausdorffLoss	24.38126589	Hard
MIOFlow	Ma	HausdorffLoss	10158.65353	Hard
MIOFlow	MaOlaniru	HausdorffLoss	15140.21268	Hard
MIOFlow	Olaniru	HausdorffLoss	8527.967862	Hard
MIOFlow	Zebrafish	HausdorffLoss	48.22885138	Hard
MIOFlow	Drosophila	HausdorffLoss	58.253998	Medium
MIOFlow	MEF	HausdorffLoss	28.55174273	Medium
MIOFlow	Ma	HausdorffLoss	17654.8955	Medium
MIOFlow	MaOlaniru	HausdorffLoss	111421837.6	Medium
MIOFlow	Olaniru	HausdorffLoss	10168.62749	Medium
MIOFlow	Zebrafish	HausdorffLoss	46.34249996	Medium
MIOFlow	Drosophila	MMDLoss	3.22E-07	Easy
MIOFlow	MEF	MMDLoss	6.03E-07	Easy
MIOFlow	Ma	MMDLoss	3.70E-09	Easy
MIOFlow	MaOlaniru	MMDLoss	6.52E-09	Easy
MIOFlow	Olaniru	MMDLoss	9.81E-09	Easy
MIOFlow	Zebrafish	MMDLoss	9.74E-07	Easy
MIOFlow	Drosophila	MMDLoss	2.76E-07	Hard
MIOFlow	MEF	MMDLoss	6.57E-07	Hard
MIOFlow	Ma	MMDLoss	4.00E-09	Hard
MIOFlow	MaOlaniru	MMDLoss	7.87E-09	Hard
MIOFlow	Olaniru	MMDLoss	1.13E-08	Hard
MIOFlow	Zebrafish	MMDLoss	1.01E-06	Hard
MIOFlow	Drosophila	MMDLoss	2.31E-07	Medium
MIOFlow	MEF	MMDLoss	6.05E-07	Medium
MIOFlow	Ma	MMDLoss	5.27E-09	Medium
MIOFlow	MaOlaniru	MMDLoss	1.05E-08	Medium
MIOFlow	Olaniru	MMDLoss	1.01E-08	Medium
MIOFlow	Zebrafish	MMDLoss	9.88E-07	Medium
MIOFlow	Drosophila	WassersteinOTLoss	0.26196318	Easy
MIOFlow	MEF	WassersteinOTLoss	0.065505276	Easy
MIOFlow	Ma	WassersteinOTLoss	4.510274863	Easy
MIOFlow	MaOlaniru	WassersteinOTLoss	227.7380023	Easy

MIOFlow	Olaniru	WassersteinOTLoss	25.18724657	Easy
MIOFlow	Zebrafish	WassersteinOTLoss	0.263699482	Easy
MIOFlow	Drosophila	WassersteinOTLoss	0.291731277	Hard
MIOFlow	MEF	WassersteinOTLoss	0.062839137	Hard
MIOFlow	Ma	WassersteinOTLoss	7.682378814	Hard
MIOFlow	MaOlaniru	WassersteinOTLoss	30.19441621	Hard
MIOFlow	Olaniru	WassersteinOTLoss	49.48846602	Hard
MIOFlow	Zebrafish	WassersteinOTLoss	0.294989285	Hard
MIOFlow	Drosophila	WassersteinOTLoss	0.346741395	Medium
MIOFlow	MEF	WassersteinOTLoss	0.076035468	Medium
MIOFlow	Ma	WassersteinOTLoss	18.54495416	Medium
MIOFlow	MaOlaniru	WassersteinOTLoss	714501080.5	Medium
MIOFlow	Olaniru	WassersteinOTLoss	60.5855388	Medium
MIOFlow	Zebrafish	WassersteinOTLoss	0.345975141	Medium
PISDE	Drosophila	EnergyDistanceLoss	0.001158326	Easy
PISDE	MEF	EnergyDistanceLoss	0.000947599	Easy
PISDE	Zebrafish	EnergyDistanceLoss	0.000708814	Easy
PISDE	Drosophila	EnergyDistanceLoss	0.001163904	Hard
PISDE	MEF	EnergyDistanceLoss	0.000748295	Hard
PISDE	Zebrafish	EnergyDistanceLoss	0.000983427	Hard
PISDE	Drosophila	EnergyDistanceLoss	0.001793639	Medium
PISDE	MEF	EnergyDistanceLoss	0.001315119	Medium
PISDE	Zebrafish	EnergyDistanceLoss	0.00405409	Medium
PISDE	Drosophila	HausdorffLoss	50.54353846	Easy
PISDE	MEF	HausdorffLoss	29.70573344	Easy
PISDE	Zebrafish	HausdorffLoss	53.91215661	Easy
PISDE	Drosophila	HausdorffLoss	50.50247884	Hard
PISDE	MEF	HausdorffLoss	30.86891908	Hard
PISDE	Zebrafish	HausdorffLoss	53.33080363	Hard
PISDE	Drosophila	HausdorffLoss	54.12108906	Medium
PISDE	MEF	HausdorffLoss	34.12158337	Medium
PISDE	Zebrafish	HausdorffLoss	58.33105933	Medium
PISDE	Drosophila	MMDLoss	3.22E-07	Easy
PISDE	MEF	MMDLoss	5.50E-07	Easy
PISDE	Zebrafish	MMDLoss	8.99E-07	Easy
PISDE	Drosophila	MMDLoss	2.76E-07	Hard
PISDE	MEF	MMDLoss	5.57E-07	Hard
PISDE	Zebrafish	MMDLoss	9.14E-07	Hard
PISDE	Drosophila	MMDLoss	2.31E-07	Medium
PISDE	MEF	MMDLoss	5.20E-07	Medium
PISDE	Zebrafish	MMDLoss	9.12E-07	Medium
PISDE	Drosophila	WassersteinOTLoss	0.337457815	Easy
PISDE	MEF	WassersteinOTLoss	0.136216972	Easy
PISDE	Zebrafish	WassersteinOTLoss	0.443934701	Easy

PISDE	Drosophila	WassersteinOTLoss	0.35945191	Hard
PISDE	MEF	WassersteinOTLoss	0.147991908	Hard
PISDE	Zebrafish	WassersteinOTLoss	0.488143181	Hard
PISDE	Drosophila	WassersteinOTLoss	0.430409532	Medium
PISDE	MEF	WassersteinOTLoss	0.185454092	Medium
PISDE	Zebrafish	WassersteinOTLoss	0.72929138	Medium
PRESCIENT	Drosophila	EnergyDistanceLoss	0.002872157	Easy
PRESCIENT	MEF	EnergyDistanceLoss	0.001179531	Easy
PRESCIENT	Ma	EnergyDistanceLoss	0.001106655	Easy
PRESCIENT	MaOlaniru	EnergyDistanceLoss	0.00295644	Easy
PRESCIENT	Olaniru	EnergyDistanceLoss	0.002554292	Easy
PRESCIENT	Zebrafish	EnergyDistanceLoss	0.005503767	Easy
PRESCIENT	Drosophila	EnergyDistanceLoss	0.003031776	Hard
PRESCIENT	MEF	EnergyDistanceLoss	0.001427212	Hard
PRESCIENT	Ma	EnergyDistanceLoss	0.001105659	Hard
PRESCIENT	MaOlaniru	EnergyDistanceLoss	0.004798147	Hard
PRESCIENT	Olaniru	EnergyDistanceLoss	0.003664834	Hard
PRESCIENT	Zebrafish	EnergyDistanceLoss	0.005492984	Hard
PRESCIENT	Drosophila	EnergyDistanceLoss	0.003272043	Medium
PRESCIENT	MEF	EnergyDistanceLoss	0.001388766	Medium
PRESCIENT	Ma	EnergyDistanceLoss	0.002063532	Medium
PRESCIENT	MaOlaniru	EnergyDistanceLoss	0.005074721	Medium
PRESCIENT	Olaniru	EnergyDistanceLoss	0.00351101	Medium
PRESCIENT	Zebrafish	EnergyDistanceLoss	0.002732439	Medium
PRESCIENT	Drosophila	HausdorffLoss	58.5115722	Easy
PRESCIENT	MEF	HausdorffLoss	27.29671253	Easy
PRESCIENT	Ma	HausdorffLoss	9976.008213	Easy
PRESCIENT	MaOlaniru	HausdorffLoss	11874.67724	Easy
PRESCIENT	Olaniru	HausdorffLoss	5793.57837	Easy
PRESCIENT	Zebrafish	HausdorffLoss	60.75464409	Easy
PRESCIENT	Drosophila	HausdorffLoss	65.08759461	Hard
PRESCIENT	MEF	HausdorffLoss	26.82511211	Hard
PRESCIENT	Ma	HausdorffLoss	11444.52802	Hard
PRESCIENT	MaOlaniru	HausdorffLoss	9573.421596	Hard
PRESCIENT	Olaniru	HausdorffLoss	6986.266022	Hard
PRESCIENT	Zebrafish	HausdorffLoss	77.97171273	Hard
PRESCIENT	Drosophila	HausdorffLoss	48.73807911	Medium
PRESCIENT	MEF	HausdorffLoss	27.14359206	Medium
PRESCIENT	Ma	HausdorffLoss	12949.12895	Medium
PRESCIENT	MaOlaniru	HausdorffLoss	7398.014326	Medium
PRESCIENT	Olaniru	HausdorffLoss	7177.1657	Medium
PRESCIENT	Zebrafish	HausdorffLoss	55.01386555	Medium
PRESCIENT	Drosophila	MMDLoss	3.22E-07	Easy
PRESCIENT	MEF	MMDLoss	7.58E-07	Easy

PRESCIENT	Ma	MMDLoss	3.17E-09	Easy
PRESCIENT	MaOlaniru	MMDLoss	5.99E-09	Easy
PRESCIENT	Olaniru	MMDLoss	9.81E-09	Easy
PRESCIENT	Zebrafish	MMDLoss	8.99E-07	Easy
PRESCIENT	Drosophila	MMDLoss	2.76E-07	Hard
PRESCIENT	MEF	MMDLoss	7.48E-07	Hard
PRESCIENT	Ma	MMDLoss	3.32E-09	Hard
PRESCIENT	MaOlaniru	MMDLoss	7.52E-09	Hard
PRESCIENT	Olaniru	MMDLoss	1.06E-08	Hard
PRESCIENT	Zebrafish	MMDLoss	9.14E-07	Hard
PRESCIENT	Drosophila	MMDLoss	2.31E-07	Medium
PRESCIENT	MEF	MMDLoss	6.69E-07	Medium
PRESCIENT	Ma	MMDLoss	5.27E-09	Medium
PRESCIENT	MaOlaniru	MMDLoss	1.05E-08	Medium
PRESCIENT	Olaniru	MMDLoss	1.01E-08	Medium
PRESCIENT	Zebrafish	MMDLoss	9.12E-07	Medium
PRESCIENT	Drosophila	WassersteinOTLoss	0.281353814	Easy
PRESCIENT	MEF	WassersteinOTLoss	0.063153598	Easy
PRESCIENT	Ma	WassersteinOTLoss	8.997884222	Easy
PRESCIENT	MaOlaniru	WassersteinOTLoss	32.5494056	Easy
PRESCIENT	Olaniru	WassersteinOTLoss	21.47462729	Easy
PRESCIENT	Zebrafish	WassersteinOTLoss	0.505394218	Easy
PRESCIENT	Drosophila	WassersteinOTLoss	0.434428345	Hard
PRESCIENT	MEF	WassersteinOTLoss	0.064678308	Hard
PRESCIENT	Ma	WassersteinOTLoss	7.281337898	Hard
PRESCIENT	MaOlaniru	WassersteinOTLoss	24.03885986	Hard
PRESCIENT	Olaniru	WassersteinOTLoss	35.05762648	Hard
PRESCIENT	Zebrafish	WassersteinOTLoss	0.676300451	Hard
PRESCIENT	Drosophila	WassersteinOTLoss	0.333404777	Medium
PRESCIENT	MEF	WassersteinOTLoss	0.069069042	Medium
PRESCIENT	Ma	WassersteinOTLoss	12.82635929	Medium
PRESCIENT	MaOlaniru	WassersteinOTLoss	42.55347189	Medium
PRESCIENT	Olaniru	WassersteinOTLoss	27.42133326	Medium
PRESCIENT	Zebrafish	WassersteinOTLoss	0.433326747	Medium
scIMF	Drosophila	EnergyDistanceLoss	0.002270967	Easy
scIMF	MEF	EnergyDistanceLoss	0.000707886	Easy
scIMF	Ma	EnergyDistanceLoss	0.000772388	Easy
scIMF	MaOlaniru	EnergyDistanceLoss	0.001449526	Easy
scIMF	Olaniru	EnergyDistanceLoss	0.001801245	Easy
scIMF	Zebrafish	EnergyDistanceLoss	0.002433453	Easy
scIMF	Drosophila	EnergyDistanceLoss	0.002160979	Hard
scIMF	MEF	EnergyDistanceLoss	0.000558081	Hard
scIMF	Ma	EnergyDistanceLoss	0.00081981	Hard
scIMF	MaOlaniru	EnergyDistanceLoss	0.001764921	Hard

scIMF	Olaniru	EnergyDistanceLoss	0.002121252	Hard
scIMF	Zebrafish	EnergyDistanceLoss	0.00227388	Hard
scIMF	Drosophila	EnergyDistanceLoss	0.002269921	Medium
scIMF	MEF	EnergyDistanceLoss	0.000524969	Medium
scIMF	Ma	EnergyDistanceLoss	0.001087436	Medium
scIMF	MaOlaniru	EnergyDistanceLoss	0.003471078	Medium
scIMF	Olaniru	EnergyDistanceLoss	0.003488925	Medium
scIMF	Zebrafish	EnergyDistanceLoss	0.002108749	Medium
scIMF	Drosophila	HausdorffLoss	42.47510557	Easy
scIMF	MEF	HausdorffLoss	21.17847212	Easy
scIMF	Ma	HausdorffLoss	7370.298587	Easy
scIMF	MaOlaniru	HausdorffLoss	9069.454008	Easy
scIMF	Olaniru	HausdorffLoss	5884.509999	Easy
scIMF	Zebrafish	HausdorffLoss	48.0421416	Easy
scIMF	Drosophila	HausdorffLoss	43.08923	Hard
scIMF	MEF	HausdorffLoss	22.47941293	Hard
scIMF	Ma	HausdorffLoss	9228.029049	Hard
scIMF	MaOlaniru	HausdorffLoss	8670.432862	Hard
scIMF	Olaniru	HausdorffLoss	8008.261937	Hard
scIMF	Zebrafish	HausdorffLoss	46.34423956	Hard
scIMF	Drosophila	HausdorffLoss	44.8530627	Medium
scIMF	MEF	HausdorffLoss	26.0290114	Medium
scIMF	Ma	HausdorffLoss	13749.34724	Medium
scIMF	MaOlaniru	HausdorffLoss	8176.447854	Medium
scIMF	Olaniru	HausdorffLoss	14828.60598	Medium
scIMF	Zebrafish	HausdorffLoss	45.59815325	Medium
scIMF	Drosophila	MMDLoss	3.22E-07	Easy
scIMF	MEF	MMDLoss	6.46E-07	Easy
scIMF	Ma	MMDLoss	3.70E-09	Easy
scIMF	MaOlaniru	MMDLoss	6.51E-09	Easy
scIMF	Olaniru	MMDLoss	9.81E-09	Easy
scIMF	Zebrafish	MMDLoss	9.75E-07	Easy
scIMF	Drosophila	MMDLoss	2.76E-07	Hard
scIMF	MEF	MMDLoss	6.90E-07	Hard
scIMF	Ma	MMDLoss	4.01E-09	Hard
scIMF	MaOlaniru	MMDLoss	7.86E-09	Hard
scIMF	Olaniru	MMDLoss	1.12E-08	Hard
scIMF	Zebrafish	MMDLoss	1.01E-06	Hard
scIMF	Drosophila	MMDLoss	2.31E-07	Medium
scIMF	MEF	MMDLoss	6.36E-07	Medium
scIMF	Ma	MMDLoss	5.27E-09	Medium
scIMF	MaOlaniru	MMDLoss	1.05E-08	Medium
scIMF	Olaniru	MMDLoss	1.01E-08	Medium
scIMF	Zebrafish	MMDLoss	9.90E-07	Medium

scIMF	Drosophila	WassersteinOTLoss	0.248514518	Easy
scIMF	MEF	WassersteinOTLoss	0.048596532	Easy
scIMF	Ma	WassersteinOTLoss	5.531480091	Easy
scIMF	MaOlaniru	WassersteinOTLoss	12.48916552	Easy
scIMF	Olaniru	WassersteinOTLoss	18.75516937	Easy
scIMF	Zebrafish	WassersteinOTLoss	0.249471731	Easy
scIMF	Drosophila	WassersteinOTLoss	0.268267381	Hard
scIMF	MEF	WassersteinOTLoss	0.048117647	Hard
scIMF	Ma	WassersteinOTLoss	6.506570731	Hard
scIMF	MaOlaniru	WassersteinOTLoss	16.99789684	Hard
scIMF	Olaniru	WassersteinOTLoss	28.59599176	Hard
scIMF	Zebrafish	WassersteinOTLoss	0.278747833	Hard
scIMF	Drosophila	WassersteinOTLoss	0.305105936	Medium
scIMF	MEF	WassersteinOTLoss	0.057739212	Medium
scIMF	Ma	WassersteinOTLoss	21.58302435	Medium
scIMF	MaOlaniru	WassersteinOTLoss	26.00687113	Medium
scIMF	Olaniru	WassersteinOTLoss	64.84327766	Medium
scIMF	Zebrafish	WassersteinOTLoss	0.324275898	Medium
scNODE	Drosophila	EnergyDistanceLoss	0.002333921	Easy
scNODE	MEF	EnergyDistanceLoss	0.001309723	Easy
scNODE	Ma	EnergyDistanceLoss	0.001588603	Easy
scNODE	MaOlaniru	EnergyDistanceLoss	0.002572646	Easy
scNODE	Olaniru	EnergyDistanceLoss	0.003043603	Easy
scNODE	Zebrafish	EnergyDistanceLoss	0.002675331	Easy
scNODE	Drosophila	EnergyDistanceLoss	0.002323613	Hard
scNODE	MEF	EnergyDistanceLoss	0.001132947	Hard
scNODE	Ma	EnergyDistanceLoss	0.002644292	Hard
scNODE	MaOlaniru	EnergyDistanceLoss	0.003833174	Hard
scNODE	Olaniru	EnergyDistanceLoss	0.003321077	Hard
scNODE	Zebrafish	EnergyDistanceLoss	0.002845028	Hard
scNODE	Drosophila	EnergyDistanceLoss	0.003895232	Medium
scNODE	MEF	EnergyDistanceLoss	0.00394785	Medium
scNODE	Ma	EnergyDistanceLoss	0.002169085	Medium
scNODE	MaOlaniru	EnergyDistanceLoss	0.00441	Medium
scNODE	Olaniru	EnergyDistanceLoss	0.007747673	Medium
scNODE	Zebrafish	EnergyDistanceLoss	0.005365819	Medium
scNODE	Drosophila	HausdorffLoss	42.90068362	Easy
scNODE	MEF	HausdorffLoss	23.55770735	Easy
scNODE	Ma	HausdorffLoss	10591.74054	Easy
scNODE	MaOlaniru	HausdorffLoss	11460.55939	Easy
scNODE	Olaniru	HausdorffLoss	5959.213098	Easy
scNODE	Zebrafish	HausdorffLoss	45.92390395	Easy
scNODE	Drosophila	HausdorffLoss	43.24727512	Hard
scNODE	MEF	HausdorffLoss	24.00297534	Hard

scNODE	Ma	HausdorffLoss	11006.56499	Hard
scNODE	MaOlaniru	HausdorffLoss	11566.29376	Hard
scNODE	Olaniru	HausdorffLoss	7966.936108	Hard
scNODE	Zebrafish	HausdorffLoss	46.54992287	Hard
scNODE	Drosophila	HausdorffLoss	53.56033413	Medium
scNODE	MEF	HausdorffLoss	32.01226472	Medium
scNODE	Ma	HausdorffLoss	16952.37566	Medium
scNODE	MaOlaniru	HausdorffLoss	8314.171401	Medium
scNODE	Olaniru	HausdorffLoss	8943.800563	Medium
scNODE	Zebrafish	HausdorffLoss	52.169824	Medium
scNODE	Drosophila	MMDLoss	3.85E-07	Easy
scNODE	MEF	MMDLoss	3.73E-06	Easy
scNODE	Ma	MMDLoss	2.78E-09	Easy
scNODE	MaOlaniru	MMDLoss	5.60E-09	Easy
scNODE	Olaniru	MMDLoss	9.81E-09	Easy
scNODE	Zebrafish	MMDLoss	6.10E-07	Easy
scNODE	Drosophila	MMDLoss	3.10E-07	Hard
scNODE	MEF	MMDLoss	3.43E-06	Hard
scNODE	Ma	MMDLoss	2.80E-09	Hard
scNODE	MaOlaniru	MMDLoss	7.26E-09	Hard
scNODE	Olaniru	MMDLoss	9.92E-09	Hard
scNODE	Zebrafish	MMDLoss	5.28E-07	Hard
scNODE	Drosophila	MMDLoss	2.54E-07	Medium
scNODE	MEF	MMDLoss	3.26E-06	Medium
scNODE	Ma	MMDLoss	5.27E-09	Medium
scNODE	MaOlaniru	MMDLoss	1.05E-08	Medium
scNODE	Olaniru	MMDLoss	1.01E-08	Medium
scNODE	Zebrafish	MMDLoss	6.58E-07	Medium
scNODE	Drosophila	WassersteinOTLoss	0.254808042	Easy
scNODE	MEF	WassersteinOTLoss	0.060895368	Easy
scNODE	Ma	WassersteinOTLoss	2.246436898	Easy
scNODE	MaOlaniru	WassersteinOTLoss	12.78225009	Easy
scNODE	Olaniru	WassersteinOTLoss	24.8579373	Easy
scNODE	Zebrafish	WassersteinOTLoss	0.258190011	Easy
scNODE	Drosophila	WassersteinOTLoss	0.270612469	Hard
scNODE	MEF	WassersteinOTLoss	0.057771968	Hard
scNODE	Ma	WassersteinOTLoss	10.69183364	Hard
scNODE	MaOlaniru	WassersteinOTLoss	28.19188195	Hard
scNODE	Olaniru	WassersteinOTLoss	26.75492385	Hard
scNODE	Zebrafish	WassersteinOTLoss	0.28930596	Hard
scNODE	Drosophila	WassersteinOTLoss	0.400998484	Medium
scNODE	MEF	WassersteinOTLoss	0.16079101	Medium
scNODE	Ma	WassersteinOTLoss	17.36119125	Medium
scNODE	MaOlaniru	WassersteinOTLoss	34.43468322	Medium

scNODE	Olaniru	WassersteinOTLoss	75.56376865	Medium
scNODE	Zebrafish	WassersteinOTLoss	0.445365425	Medium
OT-CFM	Drosophila	WassersteinOTLoss	0.292071431	Easy
OT-CFM	Drosophila	MMDLoss	3.22E-07	Easy
OT-CFM	Drosophila	HausdorffLoss	45.413051	Easy
OT-CFM	Drosophila	EnergyDistanceLoss	0.001558299	Easy
OT-CFM	Zebrafish	WassersteinOTLoss	0.250816189	Easy
OT-CFM	Zebrafish	WassersteinOTLoss	0.649272257	Medium
OT-CFM	Zebrafish	WassersteinOTLoss	0.286076687	Hard
OT-CFM	Drosophila	WassersteinOTLoss	124.3037801	Medium
OT-CFM	Drosophila	WassersteinOTLoss	0.289219884	Hard
OT-CFM	MEF	WassersteinOTLoss	0.081470152	Easy
OT-CFM	MEF	WassersteinOTLoss	5.328108617	Medium
OT-CFM	MEF	WassersteinOTLoss	0.067974987	Hard
OT-CFM	Ma	WassersteinOTLoss	3.828684965	Easy
OT-CFM	Ma	WassersteinOTLoss	578.9043315	Medium
OT-CFM	Ma	WassersteinOTLoss	12.99242799	Hard
OT-CFM	Olaniru	WassersteinOTLoss	75.00498935	Easy
OT-CFM	Olaniru	WassersteinOTLoss	919.5607287	Medium
OT-CFM	Olaniru	WassersteinOTLoss	23.09547849	Hard
OT-CFM	MaOlaniru	WassersteinOTLoss	159.4107881	Easy
OT-CFM	MaOlaniru	WassersteinOTLoss	211737446.1	Medium
OT-CFM	MaOlaniru	WassersteinOTLoss	673.0588341	Hard
OT-CFM	Zebrafish	MMDLoss	3.73E-07	Easy
OT-CFM	Zebrafish	MMDLoss	5.85E-07	Medium
OT-CFM	Zebrafish	MMDLoss	3.62E-07	Hard
OT-CFM	Drosophila	MMDLoss	2.31E-07	Medium
OT-CFM	Drosophila	MMDLoss	2.76E-07	Hard
OT-CFM	MEF	MMDLoss	5.00E-07	Easy
OT-CFM	MEF	MMDLoss	3.98E-07	Medium
OT-CFM	MEF	MMDLoss	4.49E-07	Hard
OT-CFM	Ma	MMDLoss	2.78E-09	Easy
OT-CFM	Ma	MMDLoss	5.27E-09	Medium
OT-CFM	Ma	MMDLoss	2.80E-09	Hard
OT-CFM	Olaniru	MMDLoss	9.81E-09	Easy
OT-CFM	Olaniru	MMDLoss	1.01E-08	Medium
OT-CFM	Olaniru	MMDLoss	9.92E-09	Hard
OT-CFM	MaOlaniru	MMDLoss	5.60E-09	Easy
OT-CFM	MaOlaniru	MMDLoss	1.05E-08	Medium
OT-CFM	MaOlaniru	MMDLoss	7.26E-09	Hard
OT-CFM	Zebrafish	HausdorffLoss	44.21046578	Easy
OT-CFM	Zebrafish	HausdorffLoss	115.1226886	Medium
OT-CFM	Zebrafish	HausdorffLoss	44.85399277	Hard
OT-CFM	Drosophila	HausdorffLoss	2146.618572	Medium

OT-CFM	Drosophila	HausdorffLoss	44.34789977	Hard
OT-CFM	MEF	HausdorffLoss	49.4236145	Easy
OT-CFM	MEF	HausdorffLoss	359.7779503	Medium
OT-CFM	MEF	HausdorffLoss	37.14397231	Hard
OT-CFM	Ma	HausdorffLoss	12851.55524	Easy
OT-CFM	Ma	HausdorffLoss	226769.7048	Medium
OT-CFM	Ma	HausdorffLoss	34815.20715	Hard
OT-CFM	Olaniru	HausdorffLoss	12466.85794	Easy
OT-CFM	Olaniru	HausdorffLoss	59254.07876	Medium
OT-CFM	Olaniru	HausdorffLoss	11981.5908	Hard
OT-CFM	MaOlaniru	HausdorffLoss	32853.648	Easy
OT-CFM	MaOlaniru	HausdorffLoss	25256688.98	Medium
OT-CFM	MaOlaniru	HausdorffLoss	113913.4195	Hard
OT-CFM	Zebrafish	EnergyDistanceLoss	0.001779221	Easy
OT-CFM	Zebrafish	EnergyDistanceLoss	0.001485059	Medium
OT-CFM	Zebrafish	EnergyDistanceLoss	0.001762149	Hard
OT-CFM	Drosophila	EnergyDistanceLoss	0.034315916	Medium
OT-CFM	Drosophila	EnergyDistanceLoss	0.001286522	Hard
OT-CFM	MEF	EnergyDistanceLoss	0.000541341	Easy
OT-CFM	MEF	EnergyDistanceLoss	0.006747762	Medium
OT-CFM	MEF	EnergyDistanceLoss	0.000617503	Hard
OT-CFM	Ma	EnergyDistanceLoss	0.000835455	Easy
OT-CFM	Ma	EnergyDistanceLoss	0.004284811	Medium
OT-CFM	Ma	EnergyDistanceLoss	0.002007715	Hard
OT-CFM	Olaniru	EnergyDistanceLoss	0.004800288	Easy
OT-CFM	Olaniru	EnergyDistanceLoss	0.017282281	Medium
OT-CFM	Olaniru	EnergyDistanceLoss	0.001565276	Hard
OT-CFM	MaOlaniru	EnergyDistanceLoss	0.004480651	Easy
OT-CFM	MaOlaniru	EnergyDistanceLoss	1.063582667	Medium
OT-CFM	MaOlaniru	EnergyDistanceLoss	0.008549816	Hard
Squidiff	Zebrafish	WassersteinOTLoss	4887.117876	Easy
Squidiff	Zebrafish	WassersteinOTLoss	6169.793009	Medium
Squidiff	Zebrafish	WassersteinOTLoss	6172.719818	Hard
Squidiff	Ma	WassersteinOTLoss	6112.816111	Easy
Squidiff	Drosophila	WassersteinOTLoss	5954.821555	Easy
Squidiff	Drosophila	WassersteinOTLoss	5366.402353	Medium
Squidiff	Ma	WassersteinOTLoss	6142.763858	Medium
Squidiff	Drosophila	WassersteinOTLoss	6431.218438	Hard
Squidiff	MEF	WassersteinOTLoss	5640.033965	Easy
Squidiff	Ma	WassersteinOTLoss	6084.968694	Hard
Squidiff	MEF	WassersteinOTLoss	6184.290348	Medium
Squidiff	MEF	WassersteinOTLoss	5324.083472	Hard
Squidiff	MaOlaniru	WassersteinOTLoss	6166.045861	Easy
Squidiff	Olaniru	WassersteinOTLoss	6168.09419	Easy

Squidiff	MaOlaniru	WassersteinOTLoss	6173.57081	Medium
Squidiff	Olaniru	WassersteinOTLoss	6187.175473	Medium
Squidiff	MaOlaniru	WassersteinOTLoss	6160.177525	Hard
Squidiff	Ma	MMDLoss	2.78E-09	Easy
Squidiff	Ma	MMDLoss	5.27E-09	Medium
Squidiff	Ma	MMDLoss	2.80E-09	Hard
Squidiff	MaOlaniru	MMDLoss	5.60E-09	Easy
Squidiff	MaOlaniru	MMDLoss	1.05E-08	Medium
Squidiff	MaOlaniru	MMDLoss	7.26E-09	Hard
Squidiff	Ma	HausdorffLoss	24186.99711	Easy
Squidiff	Ma	HausdorffLoss	25048.65702	Medium
Squidiff	Ma	HausdorffLoss	23644.61727	Hard
Squidiff	MaOlaniru	HausdorffLoss	28076.63589	Easy
Squidiff	MaOlaniru	HausdorffLoss	23039.56956	Medium
Squidiff	MaOlaniru	HausdorffLoss	24150.95035	Hard
Squidiff	Ma	EnergyDistanceLoss	0.254909009	Easy
Squidiff	Ma	EnergyDistanceLoss	0.252412055	Medium
Squidiff	Ma	EnergyDistanceLoss	0.252713519	Hard
Squidiff	MaOlaniru	EnergyDistanceLoss	0.251490713	Easy
Squidiff	MaOlaniru	EnergyDistanceLoss	0.23667243	Medium
Squidiff	MaOlaniru	EnergyDistanceLoss	0.245970147	Hard
Squidiff	Olaniru	WassersteinOTLoss	6182.073739	Hard
Squidiff	Zebrafish	MMDLoss	3.73E-07	Easy
Squidiff	Zebrafish	MMDLoss	5.85E-07	Medium
Squidiff	Zebrafish	MMDLoss	3.62E-07	Hard
Squidiff	Drosophila	MMDLoss	3.22E-07	Easy
Squidiff	Drosophila	MMDLoss	2.31E-07	Medium
Squidiff	Drosophila	MMDLoss	2.76E-07	Hard
Squidiff	MEF	MMDLoss	5.00E-07	Easy
Squidiff	MEF	MMDLoss	3.98E-07	Medium
Squidiff	MEF	MMDLoss	4.49E-07	Hard
Squidiff	Olaniru	MMDLoss	9.81E-09	Easy
Squidiff	Olaniru	MMDLoss	1.01E-08	Medium
Squidiff	Olaniru	MMDLoss	9.92E-09	Hard
Squidiff	Zebrafish	HausdorffLoss	6166.883587	Easy
Squidiff	Zebrafish	HausdorffLoss	5365.949519	Medium
Squidiff	Zebrafish	HausdorffLoss	5423.699676	Hard
Squidiff	Drosophila	HausdorffLoss	7827.546289	Easy
Squidiff	Drosophila	HausdorffLoss	5159.703523	Medium
Squidiff	Drosophila	HausdorffLoss	7116.232411	Hard
Squidiff	MEF	HausdorffLoss	6942.228705	Easy
Squidiff	MEF	HausdorffLoss	5397.325175	Medium
Squidiff	MEF	HausdorffLoss	6035.144866	Hard
Squidiff	Olaniru	HausdorffLoss	21972.84963	Easy

Squidiff	Olaniru	HausdorffLoss	23259.99731	Medium
Squidiff	Olaniru	HausdorffLoss	23247.55989	Hard
Squidiff	Zebrafish	EnergyDistanceLoss	0.882440451	Easy
Squidiff	Zebrafish	EnergyDistanceLoss	1.021993245	Medium
Squidiff	Zebrafish	EnergyDistanceLoss	1.021151353	Hard
Squidiff	Drosophila	EnergyDistanceLoss	0.997539825	Easy
Squidiff	Drosophila	EnergyDistanceLoss	0.950077737	Medium
Squidiff	Drosophila	EnergyDistanceLoss	1.048989948	Hard
Squidiff	MEF	EnergyDistanceLoss	0.985500828	Easy
Squidiff	MEF	EnergyDistanceLoss	1.028826566	Medium
Squidiff	MEF	EnergyDistanceLoss	0.957994155	Hard
Squidiff	Olaniru	EnergyDistanceLoss	0.239015959	Easy
Squidiff	Olaniru	EnergyDistanceLoss	0.239391119	Medium
Squidiff	Olaniru	EnergyDistanceLoss	0.238559678	Hard

Supplementary Table S3: ARI-specific Embedding Coherence Results

Method	Dataset	ARI (ground truth)	ARI (projected)	ARI (Gex+PCA ground truth)	ARI (Gex+PCA projected)
OT-CFM	Suo	0.331669491	0.311094026	0.331290724	0.338766374
OT-CFM	GarciaAlonso	0.218329087	0.179904821	0.212617365	0.179065969
OT-CFM	Ma	0.151140969	0.173073196	0.149066489	0.181021609
MIOFlow	Suo	0.000229359	0.008553244	0.324335373	0.009375081
MIOFlow	GarciaAlonso	0.003200697	0.009390837	0.216921054	0.018735621
MIOFlow	Ma	0.008533472	0.008746611	0.167166533	0.010248588
scNODE	Suo	0.38192706	0.534581309	0.33318741	0.275056025
scNODE	GarciaAlonso	0.184504279	0.238745152	0.216195116	0.098024517
scNODE	Ma	0.139674866	0.13451747	0.148895103	0.078166157
CellMNN	Suo	0.308363514	0.328396192	0.330304225	0.270382673
CellMNN	GarciaAlonso	0.230168311	0.264471161	0.21779393	0.204706715
CellMNN	Ma	0.132842598	0.175620956	0.148605613	0.197519442
PISDE	Suo	0.000196229	0.008219985	0.338929317	0.007542427
PISDE	GarciaAlonso	0.003062335	0.007156075	0.213564389	0.016639894
PISDE	Ma	0.00804364	0.011806589	0.147273257	0.001192374
Squidiff	Suo	0.388533842	0.420583699	0.318084883	0.027738008
Squidiff	GarciaAlonso	0.221542621	0.384525646	0.218380342	0.000133054
Squidiff	Ma	0.172335581	0.086889344	0.147689002	0
scIMF	Suo	0.000899581	0.022087241	0.350075789	0.041458473
scIMF	GarciaAlonso	0.005494306	0.026573567	0.25140012	0.070562175
scIMF	Ma	0.010470396	0.011553396	0.163256123	0.011696532
PRESCIENT	Suo	0.000218814	0.008127245	0.349313152	0.013748698
PRESCIENT	GarciaAlonso	0.003422174	0.007394008	0.215163546	0.020991611
PRESCIENT	Ma	0.004479957	0.013612891	0.148675717	0.027857897

Supplementary Table S4: Entropy-specific Embedding Coherence results

Method	Dataset	Entropy (Ground Truth)	Entropy (Projected)
OT-CFM	Suo	0.240246104	0.328296159
OT-CFM	GarciaAlonso	0.299479375	0.470890849
OT-CFM	Ma	0.378152995	0.445756364
MIOFlow	Suo	0.798301457	0.768143579
MIOFlow	GarciaAlonso	0.638706347	0.638039166
MIOFlow	Ma	0.433187603	0.439086733
scNODE	Suo	0.292287157	0.299780514
scNODE	GarciaAlonso	0.458425206	0.512474985
scNODE	Ma	0.274430046	0.379502305
CellMNN	Suo	0.202851437	0.176084615
CellMNN	GarciaAlonso	0.193163709	0.140041076
CellMNN	Ma	0.230808882	0.129509339
PISDE	Suo	0.800791627	0.775204891
PISDE	GarciaAlonso	0.640980693	0.649573218
PISDE	Ma	0.424935893	0.453877603
Squidiff	Suo	0.230682539	0.700958085
Squidiff	GarciaAlonso	0.284640794	0.794694317
Squidiff	Ma	0.315115384	0.628981379
scIMF	Suo	0.798428016	0.767701642
scIMF	GarciaAlonso	0.637599337	0.636920972
scIMF	Ma	0.429973078	0.438781344
PRESCIENT	Suo	0.801340231	0.775994524
PRESCIENT	GarciaAlonso	0.642756559	0.652196436
PRESCIENT	Ma	0.433078752	0.453652698
